# Identifying Activated T Cells in Reconstituted RAG Deficient Mice Using Retrovirally Transduced Pax5 Deficient Pro-B Cells

**DOI:** 10.1371/journal.pone.0005115

**Published:** 2009-04-02

**Authors:** Nadesan Gajendran, Dominique Vanhecke, Volker Brinkmann, Stefan H. E. Kaufmann

**Affiliations:** 1 Department of Immunology, Max Planck Institute for Infection Biology, Berlin, Germany; 2 Department Vertebrate Genomics, Max Planck Institute for Molecular Genetics, Berlin, Germany; Charité-Universitätsmedizin Berlin, Germany

## Abstract

Various methods have been used to identify activated T cells such as binding of MHC tetramers and expression of cell surface markers in addition to cytokine-based assays. In contrast to these published methods, we here describe a strategy to identify T cells that respond to any antigen and track the fate of these activated T cells. We constructed a retroviral double-reporter construct with enhanced green fluorescence protein (EGFP) and a far-red fluorescent protein from *Heteractis crispa* (HcRed). LTR-driven EGFP expression was used to enrich and identify transduced cells, while HcRed expression is driven by the CD40Ligand (CD40L) promoter, which is inducible and enables the identification and cell fate tracing of T cells that have responded to infection/inflammation. Pax5 deficient pro-B cells that can give rise to different hematopoietic cells like T cells, were retrovirally transduced with this double-reporter cassette and were used to reconstitute the T cell pool in RAG1 deifcient mice that lack T and B cells. By using flow cytometry and histology, we identified activated T cells that had developed from Pax5 deficient pro-B cells and responded to infection with the bacterial pathogen *Listeria monocytogenes*. Microscopic examination of organ sections allowed visual identification of HcRed-expressing cells. To further characterize the immune response to a given stimuli, this strategy can be easily adapted to identify other cells of the hematopoietic system that respond to infection/inflammation. This can be achieved by using an inducible reporter, choosing the appropriate promoter, and reconstituting mice lacking cells of interest by injecting gene-modified Pax5 deficient pro-B cells.

## Introduction

Characterization of the immune response is central to an understanding of processes underlying infection/inflammation. Most strategies have attempted to measure CD8^+^ T cell responses. They include staining with peptide-loaded major histocompatibility complex (MHC) tetramers [1] intracellular cytokine staining combined with surface marker staining by flow cytometry or ELISpot analysis following antigen stimulation [2] and genetic tagging in transgenic mice using the Cre/lox strategy [3], [4].

Tetramer technology is a useful tool in flow cytometric studies. Combined with cell surface and intracellular phenotyping, it is highly specific and sensitive. However, this technique does not lend itself to histology and only single specificities can be analyzed. Cytokine-based techniques allow the simultaneous analysis of immune responses to several antigenic epitopes, but have the disadvantage of being indirect and prone to variability. More recently, the use of transgenic mice in which activated CD8^+^ T cells are tagged utilizing the Cre/lox strategy [3], [4], has allowed characterization of activated CD8^+^ T cells. However as this approach relies on expression of Cre by a specific promoter, it is irreversible. Indeed, Cre expression, even if transiently expressed during development or due to endogenous infections, results in permanently tagged CD8^+^ T cells upon lox P recombination. Consequently, tagged CD8^+^ T cells could represent a pool of cells, including cells where Cre was transiently expressed during development or during intrathymic selection; T cells responding to antigens from gut flora or nutrients; and T cells responding to the infection/inflammation under investigation.

Notably, these cumulative effects can impair characterization of memory CD8^+^ T cells as they would represent a fraction of all activated and non-activated CD8^+^ T cells rather than those T cells that specifically responded to infection/inflammation under investigation.

Here we describe a strategy to identify activated T cells, utilizing Pax5^−/−^ pro-B cells retrovirally transduced with a double-reporter cassette to reconstitute the T cell pool in RAG1^−/−^ mice. This strategy not only allows T cell responses to be analyzed irrespective of antigen specificity, but also permits identification of all activated T cells responding to infection/inflammation. Pax5^−/−^ pro-B cells have a developmental block at the early pro-B cell stage but retain self-renewal and lymphomyeloid potential and, with different efficiencies, give rise to almost all cells of the hematopoietic system with the exception of B cells [5]–[9]. Using gene-modified Pax5^−/−^ cells to reconstitute the T cell pool in RAG1^−/−^ mice, we achieved a degree of specificity for monitoring T cell activation superior to that achieved by, for example, staining for CD40L expression upon infection in wild type mice. In the latter case, not only T cells but any cell that expresses CD40L is stained and assessment depends on specificity of the antibody used against CD40L. In the strategy used here, changing the promoter driving HcRed expression and reconstituting mice lacking a specific hematopoietic cell by injecting gene-modified Pax5^−/−^ pro-B cells, allows the identification and cell fate tracing of those reconstituted cells responding to defined stimuli.

## Results

### Reconstitution of the T cell pool in RAG1^−/−^ mice

**Figure 1:**
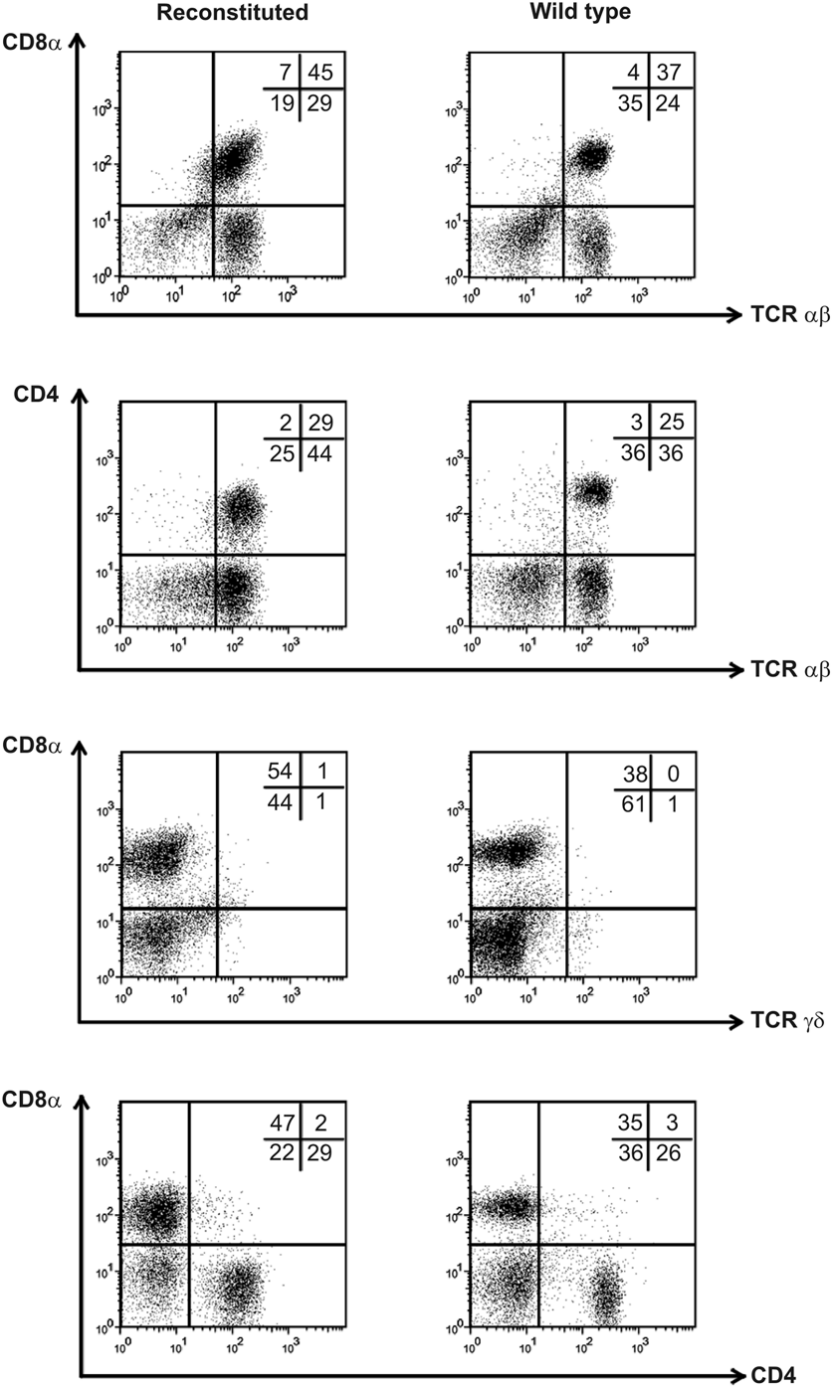
Pax5^−/−^ cells reconstitutes mature T cells in RAG1^−/−^ mice. Flow cytometric analysis of cells isolated from (axial) lymph nodes from either RAG1^−/−^ mice, which received 5×10^5^ Pax5^−/−^ cells intravenously, 4 weeks earlier or from wild type C57Bl/6 mice.. Lymph node cells were stained for CD4, CD8 and/or TCRαβ or TCRγδ. These results demonstrate that the mature T cell pool in the reconstituted RAG1^−/−^ mice is comparable to that of wild type mice. This experiment was performed twice.

As a first step, we reconstituted the T cell pool in Rag1^−/−^ mice with gene-modified Pax5^−/−^ cells. These cells, when injected intravenously into the lateral tail vein of irradiated RAG1^−/−^ mice (on a C57Bl/6 background), reconstitute the T cell pool [8]. Figure 1 shows that T cells, which developed in these mice, express T cell receptor (TCR) α/β chains or γ/δ chains and were either mature CD4 or CD8 single positives (sp), comparable to T cells in wild type mice. In auxiliary lymph nodes, the reconstituted mice contained 45% CD8 TCR α/β and 1.0% CD8 negative TCR γ/δ as compared to 37% and 1.0%, respectively, in wildtype (wt) mice (Fig. 1).

### Retroviral transduction of Pax5^−/−^ pro-B cells

**Figure 2:**
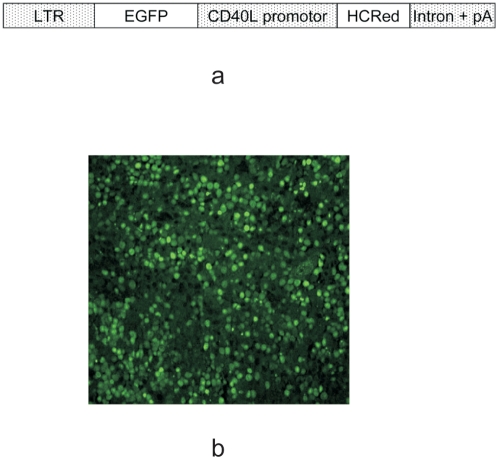
Gene-modified Pax5^−/−^ cells reconstitutes EGFP^+^ T cell pool in RAG1^−/−^ mice. (a) Schematic drawing of the double-reporter retroviral construct used for transduction of Pax5^−/−^ pro-B cells. (b) Histological examination of a spleen section from a RAG1^−/−^ mouse reconstituted with retrovirally transduced Pax5^−/−^ cells that expressed EGFP constitutively. EGFP is mainly detected in the nuclei of cells. This experiment was performed twice.

We chose the CD40L promoter to drive HcRed expression as this promoter activity is largely restricted to activated T cells and a LTR promoter to drive the EGFP reporter, in order to select and enrich the transduced cells. Pax5^−/−^ pro-B cells were transduced with the retroviral double-reporter construct (Fig. 2a) and sorted three times with a cell culture phase between sortings to achieve a homogeneous population of cells that express EGFP. Reconstitution of RAG1^−/−^ mice with the EGFP^+^ Pax5^−/−^ pro-B cells resulted in a T cell pool containing integrated retroviral construct and expressing EGFP. Histological sections of the spleen from these reconstituted RAG1^−/−^ mice revealed EGFP-expressing cells (Fig. 2b), with EGFP being localized to the nucleus.

### Identification of HcRed-expressing cells

**Figure 3:**
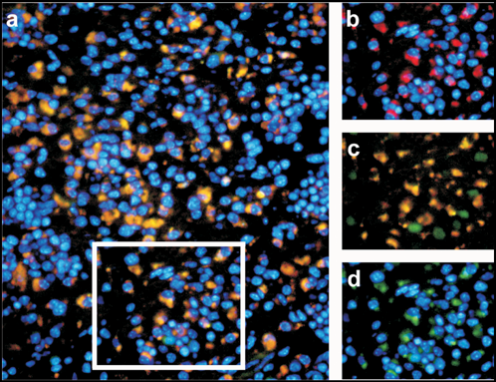
HcRed expression in reconstituted T-cell pool. (a) HcRed expression in cells of a spleen section from a mouse subjected to infection with *Listeria monocytogenes* for 2 days. (b–d) Detailed view of the boxed section. (b) HcRed expression, (c) combined EGFP and HcRed expression and (d) EGFP expression only. This experiment was performed twice.

Reconstituted Rag1^−/−^ mice were infected with *Listeria monocytogenes* and analyzed 2 days later for the presence of activated cells in spleen sections as identified by CD40L-driven HcRed expression in the cytoplasm (Fig. 3a,b). In contrast to EGFP the tetrameric HcRed complex is excluded from the nucleus and exclusively localizes to the cytoplasm. Upon HcRed expression, EGFP expression is downregulated (Fig. 3c) as evidenced by the absence of green fluorescence in the nucleus of HcRed^+^ cells. This results from the fact that once active, the CD40L promoter interferes at the transcriptional level with expression of the upstream EGFP gene driven by the retroviral LTR. Activation of T cells following *L. monocytogenes* infection was specific since not all cells in the spleen became HcRed^+^,as shown by nuclear counter-staining (Fig. 3b). Cells in the infected mice, where EGFP was observed in the nucleus (Fig. 3c) were non-activated T cells as they did not express HcRed, which is under the control of the CD40L promoter. In fact, the weak green fluorescence signals seen in the cytoplasm of cells in Figure 3d are not cells expressing the EGFP gene. Indeed, these cells were initially not visible when looking for EGFP but appeared rapidly following laser exposure of the tissue sections during microscopy. Similar red-to-green photoconversions were also reported for dsRED fluorescent proteins [10] and result from conformational changes in HcRed following exposure to high energy radiation. Fluorescence signals from EGFP can be distinguished from the green fluorescence that is simultaneously emitted by HcRed proteins based on the subcellular localisation of the signals as EGFP proteins mainly locates to the nucleus and HcRed proteins are restricted to the cytoplasm.

Indeed the few cells in Figure 3c that display a green fluorescence in the nucleus but not in the cytoplasm were also HcRed negative. In contrast, cells which expressed HcRed displayed red and green fluorescence in the cytoplasm only, corresponding with cytoplasmic localisation of HcRed proteins.

## Discussion

Here we report a strategy for the *in situ* identification of activated T cells based on the transduction of Pax5^−/−^ pro-B cells. By using a double-reporter system, transduced cells could be enriched using flowcytometry based on a constitutively expressed EGFP gene. These enriched cells were then used to reconstitute the T-cell pool in T cell deficient RAG1^−/−^ mice, thus increasing specificity and concomitantly reducing background signals during *in situ* detection of activated T cells. Indeed, Pax5^−/−^ pro-B cells were shown to give rise to other hematopoietic cells beside T cells, such as dendritic and NK cells. However, these cells are unlikely to develop in RAG1^−/−^ mice due to competition with already established endogenous cells of the same lineage. Although CD40L expression has been reported on cells other than activated T cells the preferential development of T cells from Pax5^−/−^ pro-B cells in RAG1^−/−^ mice means that HcRed expression by the double-reporter system specifically reports the presence of activated T cells only.

In contrast to the Cre/lox strategy published by others [3], [4] where all activated cells are permanently tagged, our strategy is specific: cells where the CD40L promoter may have been active at an earlier time point do not contribute to the pool of cells expressing HcRed during infection. Indeed, HcRed expression would have been downregulated in such cells in absence of a continued stimulus. Hence only activated T cells recently responding to an infection will show HcRed expression.

Although we demonstrated qualitative identification of responding cells on the basis of the double-reporter strategy, a quantitative analysis of cell populations expressing HcRed can be achieved using a flow cytometer equipped with an appropriate laser to detect cells expressing HcRed. Alternatively other combinations of fluorescent proteins can be used allowing multi-parameter cytometry.

The double-reporter construct is designed such that when the CD40L promoter is active, there is transcriptional interference of the LTR-driven EGFP expression. This interference results in EGFP-specific transcripts without a polyadenylation signal, which consequently becomes rapidly degraded. In contrast, transcription initiated at the CD40L promoter results in stable transcripts that terminate with a polyadenylation signal. When CD40L promoter is inactive, LTR-driven transcription terminates at the end of the construct (Fig. 2a) and is polyadenylated resulting in stable mRNA. In the absence of an internal ribosome entry site (IRES) upstream of HcRed, only EGFP will be translated from these transcripts. As EGFP was initially used to sort cells containing the retroviral construct, its subsequent expression or lack thereof does not affect identification of HcRed-expressing cells. In fact, the presence of those few cells showing EGFP in the nuclei and absence of HcRed in cytoplasm improves discrimination between activated and non-activated transduced cells and furthermore confirms that expression of HcRed is not leaky.

The strategy presented here, when combined with *in vivo* two-photon microscopy of lymphoid organs [11] offers a powerful tool for the detailed analysis of infection-induced T cell activation, This includes dynamics and site/location of activation and allows the analysis of interaction of activated cells with other cells of the immune system.

In addition, since Pax5^−/−^ pro-B cells can give rise to many other hematopoietic lineages, the strategy used in this study can be modified to identify different cells of the hematopoietic system responding to infection, and is only limited by the availability of the appropriate promoter and availability of mice lacking cells of interest.

In summary, we have used gene modified Pax5^−/−^ cells, which retain self-renewal and lymphomyeloid potential, to reconstitute the T cell pool in T and B cell deficient Rag1^−/−^ mice. The double reporter retroviral construct, in retrovirally transduced Pax5^−/−^ cells, allowed the identification of transduced cells through constitutive EGFP expression and the identification and cell fate tracing of activated T cells that responded to infection through the CD40L driven HcRed expression. This strategy can be easily adapted to identify other cells of the hematopoietic system with an inducible reporter, by choosing the appropriate promoter and reconstituting mice lacking cells of interest by injecting gene-modified Pax5^−/−^ pro-B cells.

## Materials and Methods

### Ethics Statement

Mice were bred in our animal facility at the Federal Institute for Health Protection of Consumers and Veterinary Medicine (Berlin, Germany) and experiments were conducted according to the German Animal Protection Law.

### *L. monocytogenes* infection of mice

C57Bl/6 and RAG1^−/−^ mice on a C57Bl/6 background were used for this study. *L. monocytogenes* strain EGD was injected into the lateral tail vein in a volume of 200 µl of PBS (Gibco) containing about 5×10^3^ colony forming units (CFU). Bacteria were grown in tryptic soy broth (TSB) overnight, washed twice in PBS and then resuspended in PBS at 2.5×10^4^ CFU per ml.

### Pro-B cell culture

B220^+^ pro-B cells sorted from the bone marrow of Pax5^−/−^ mice were a kind gift from Antonius G. Rolink (University of Basel) [12]. They were cultured on γ-irradiated ST2 stromal cells in IL-7-containing RPMI medium (Gibco) as described earlier [13].

### Generation of EGFP^+^ HcRed^+^ pro-B cells

The retroviral vector LXSP (kind gift from A.G. Rolink, Basel) was used to generate the double-reporter vector. EGFP was cloned downstream of the retroviral LTR promoter followed by the CD40L promoter driving the expression of HcRed (Clontech) with the small t-intron and polyadenylation sequence from SV40 (Fig. 2a). Consequently, cells transfected with this construct will constitutively express EGFP and will only express HcRed, a far-red fluorescent protein from *Heteractis crispa*, upon activation of CD40L promoter, e.g. upon antigen challenge. The vector was transfected into the ecotrophic cells, ψ2mp34 (kind gift from Ryoichiro Kageyama, Kyoto), which produced the packaged retrovirus. EGFP^+^ ψ2mp34 cells were then sorted and cultured. As EGFP is expressed constitutively, this enabled the selection of cells that contained stably integrated viral DNA. The cell sorting and cell culture phase was repeated three times.

Pax5^−/−^ pro-B cells were co-cultured on irradiated ψ2mp34 EGFP^+^ cells in the presence of IL-7. After 2 days, the pro-B cells were harvested by centrifugation and cultured on freshly irradiated ST-2 stroma cells. These pro-B cells were then sorted for EGFP^+^ cells. The cell sorting and culture phase was repeated three times resulting in 100% EGFP^+^ Pax5^−/−^ pro-B cells. The transduced Pax5^−/−^ pro-B cells were then used for reconstituting the T cell pool in RAG1^−/−^ mice.

### Flow cytometry and cell sorting

PE, FITC, APC, and biotin-conjugated mAb specific for CD4, CD8, TCRα/β and TCR γ/δ were purchased from Pharmingen, BD Biosciences. Staining of cells was performed as described [14] using final dilutions of the mAb as indicated by the manufacturer. Flow cytometry was performed using the LSRII (BD Biosciences) and data were analyzed using FACS Diva software (BD Biosciences). The MOFLO (DAKO cytomation) was used for cell sorting using summit software. To obtain a pure EGFP^+^ population, cells were sorted at least three times with a cell culture phase (4–5 passages) between each sort.

### T cell reconstitution

Pax5^−/−^ pro-B cells cultured on an irradiated ST-2 stromal layer were harvested by centrifugation in 50-ml Falcon tubes (Sarstedt, Nümbrecht) at 1,500 rpm, for 5 min. Cells were then washed and resuspended in PBS at 5×10^6^/200 µl. RAG1^−/−^ mice irradiated with 500rad in a Provit 5260 machine (STS Steuerungstechnik & Stahlenschutz GmbH, Braunschweig, Germany) 1 day before, received 5×10^6^ control pro-B cells or gene-modified pro-B cells in the lateral tail vein.

### Histology

Tissue from wild type and reconstituted Rag1^−/−^ mice pre- and post-infection were fixed in 4% paraformaldehyde (PFA), dehydrated in cold acetone and embedded in Kulzer Technovit 8100 (Hereaus Kulzer, Wehrheim, Germany) at 4°C following manufacturer's instructions. After polymerization at this temperature, 3-µm sections were cut using a rotation microtome (Leica, Bensheim, Germany) and analyzed with a Leica DMRB microscope equipped with a Nikon DXM 1200 camera. For EGFP detection we used L5 filters with a maximum excitation band pass (BP) of 480/40 dichroic 505 and maximum emission BP of 527/30. For HcRed we used a Y3 filter with a maximum excitation BP of 535/50 dichroic RKP 565 and maximum emission BP of 610/75.
